# iOBPdb A Database for Experimentally Determined Functional Characterization of Insect Odorant Binding Proteins

**DOI:** 10.1038/s41597-023-02195-y

**Published:** 2023-05-19

**Authors:** Shalabh Shukla, Oliver Nakano-Baker, Dennis Godin, Devin MacKenzie, Mehmet Sarikaya

**Affiliations:** University of Washington, Seattle, WA USA

**Keywords:** Protein databases, Entomology, Proteins

## Abstract

Odorant binding proteins (OBPs) are extra-cellular proteins that solubilize and transport volatile organic compounds (VOCs). Thousands of OBPs have been identified through genome sequencing, and hundreds have been characterized by fluorescence ligand binding assays in individual studies. There is a limited understanding of the comparative structure-function relations of OBPs, primarily due to a lack of a centralized database that relates OBP binding affinity and structure. Combining 181 functional studies containing 382 unique OBPs from 91 insect species, we present a database, iOBPdb, of OBP binding affinities for 622 individual VOC targets. This initial database provides powerful search and associative capabilities for retrieving and analyzing OBP-VOC binding interaction data. We have validated this dataset using phylogenetic mapping to determine the authenticity of the collected sequences and whether they cluster according to their assigned subfamilies. Potential applications include developing molecular probes for biosensors, novel bioassays and drugs, targeted pesticides that inhibit VOC/OBP interactions, and understanding odor sensing and perception in the brain.

## Background & Summary

**Fig. 1:**
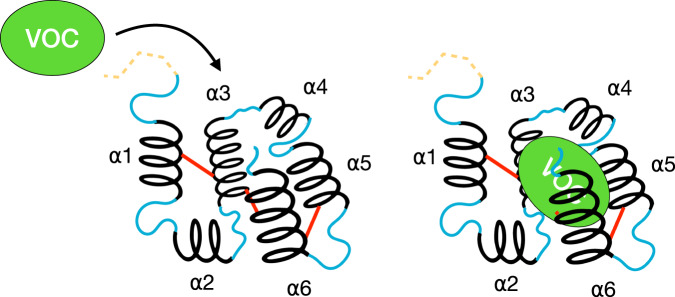
Functional domains of an odorant binding protein, OBP, relevant in capturing a volatile organic compound, VOC. In green is the VOC, in dashed yellow is the signal peptide, which is present in the nascent form of the OBP but is cleaved before it enters the extracellular environment; in solid black are alpha helices (denoted as α1-α6); in solid blue are interspersed amino acid stretches connecting the alpha helices, and in solid red are the disulfide bonds holding the OBP together. A vacant OBP is shown in panel A, and a bound OBP with a VOC is shown in Panel B. The bound OBP transports the VOC cargo inside the organism toward the sensory neural cells.

Odorant binding proteins (OBPs) are a diverse family of small, 10–20 kDa, soluble extracellular target-binding proteins found in terrestrial vertebrates and invertebrates^1,2^. Since their initial discovery in insects’ sensillum lymph in 1981^1^, thousands of new OBPs have been identified and isolated through genome sequencing and molecular biology approaches. These studies indicate no shared homology between insect and mammalian OBPs. Mammalian OBPs consist of a beta-barrel type structure, whereas the insect OBPs consist of a globular structure of alpha helices. Although OBPs are multifaceted in terms of their potential roles in both insects and mammals alike, they are primarily thought to act as odor transporters, solubilizing volatile organic compounds (VOCs) and pheromones from the surrounding air into the aqueous phase of the odor-sensory organ, such as the mucus in the nose or sensillum lymph of an antennae^2^. Figure 1 shows the typical structure of an insect OBP and how the VOC interacts with the functional domains of insect OBPs.

The primary function of OBPs is to transport odorants to sensing neurons coated with membrane-bound integral membrane olfactory receptor (OR) proteins, which recognize and bind to specific odors, thus signaling an olfactory response^2^. Insect and mammalian OR proteins also have no shared homology. Mammalian ORs are G protein-coupled receptors (GPCRs) containing seven transmembrane α helices that trigger a neural response via a second messenger cascade^3^. Insect ORs are ligand-gated ion channels comprised of two subunits: the highly variable odor-sensing OR subunit and the conserved co-receptor Orco subunit. Together they assemble into a heteromeric complex that triggers neural response directly through ion influx^4^. In addition to their primary function as odorant transport, OBPs are also involved in immunity, mating, moisture detection, signaling molecule transport, and biochemical inhibition. OBPs are expressed almost ubiquitously across organs in insects, not just the olfactory organs^5^.

A variety of naming schemes are used to describe OBP-related proteins, including chemosensory proteins (CSPs), pheromone binding proteins (PBPs), antennal-specific proteins (ASPs), antennal binding proteins (ABPXs), and others. Odorant binding proteins are often sub-categorized as either being: classic odorant binding proteins (classic OBPs), general odorant binding proteins (GOBPs), atypical odorant binding proteins (atypical OPBs), minus-C odorant binding proteins (minus-C OBPs), or plus-C odorant binding proteins (plus-C OBPs). General odorant binding proteins (GOBPs) are defined based on the ubiquitous nature of their expression in both male and female insects. PBPs are a subfamily of OBPs identified initially due to their preferential binding to pheromones in radio-labeled ligand binding studies in the early 1990s^6^. CSPs are smaller than other OBPs, share minimal sequence homology, if any, and typically only contain 4 cysteines instead of the classic 6 cysteines^7^. CSPs are primarily produced in the chemosensory organs of insects instead of the antennae^5^.

**Fig. 2:**
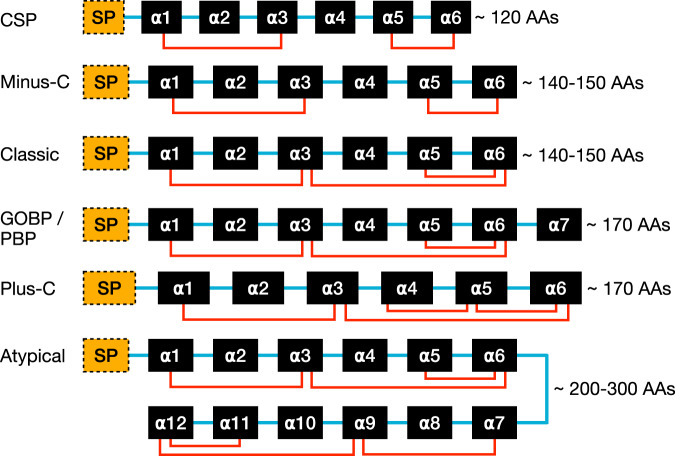
The functional domains of OBPs and their structures. The structural depiction of a classical OBP is with six alpha helices (denoted in black as α1- α6) and is held in place by three disulfide bonds (shown in red). The alpha helices are linked by short sequences with no canonical structure (shown in blue). The signaling peptide (represented as SP in yellow) at the start of the sequence marks the protein for excretion by the cell and is cleaved before entering the extracellular environment. Different arrangements of OBPs differ regarding the number of amino acids (AAs), the number of cysteine bonds, and the number of alpha helices. Note that due to the immense diversity of OBPs, many OBPs may exhibit conformations not shown in this figure.

Specific modes of distinguishing these proteins from one another include insect tissue localization, sexual dimorphic expression, insect species of discovery, species of origin of VOC (plant or predator), cysteine counts in the underlying amino acid sequence of the binding protein, alpha helices present in the folded protein, and preference of binding proteins to certain chemicals or functional groups. While insect OBPs share the same globular structure, as shown in Fig. 1, insect OBPs differ in conformation, size, and rigidity due to variations in the underlying amino acid sequences, alpha helix count, and cysteine count, as shown in Fig. 2. The classic insect OBP contains 6 highly conserved cysteine residues, which form 3 disulfide bonds. However, there are insect OBP variants with fewer than 6 cysteines that only form 2 disulfide bonds and are aptly named minus-C OBPs. Conversely, insect OBPs with 8 or more cysteines are termed plus-C OBPs. A special variant of plus-C OBPs is atypical OBPs, typically more than 20–30 amino acids longer than regular plus-C OBPs and often containing 10 or more cysteines. Atypical OBPs are sometimes called two-domain or dimer OBPs, which refer to a fusion protein of two OBPs^8^. This is not to be confused with OBP dimers, which are two distinct OBP proteins (two of the same or two different proteins) that pair to sandwich a ligand. The variation in cysteine counts drastically changes the underlying protein folding, survivability in the extracellular environment, and definition of ligand binding pocket.

The divergent molecular conformations of insect OBPs alter their ability to bind to various odorants and pheromones. While it is generally agreed that OBPs share specificity for many different molecules, several insect OBPs prefer specific molecular moieties and functional groups. Perhaps the most widely known example is LUSH, also known as DmelOBP76a, which was shown to be essential in recognizing the pheromone 11-*cis* vaccenyl acetate^9^. In a study by Xu *et al*.^9^, other OBPs could not compensate for suppressed signal transduction when LUSH was knocked out. (There remains some dispute regarding the essentiality and mechanism of action of DmelOBP76a for 11-*cis* vaccenyl acetate detection, though the enhanced affinity of LUSH for 11-*cis* vaccenyl acetate compared to other OBPs is not disputed^10^). The double domain features of atypical OBPs are also speculated to help ensnare signaling molecules, preventing signaling action, or are potentially needed for binding to unusually large ligands^11^. Conversely, fewer cysteines appear responsible for CSPs’ relatively broad binding capabilities to various ligands.

High-throughput sequencing of novel insect genomes has made it much easier to identify, isolate, and characterize novel OBPs. The binding of insect OBPs to various molecules has been widely studied using 1-N-phenylnaphthylamine (1-NPN) competitive fluorescent ligand binding assays^12^. 1-NPN is a reporter molecule that provides a continuous baseline signal as it binds to the OBP of interest^13^. Other reporter molecules, such as 1-aminoanthracene (1-AMA), are used to study the binding of mammalian OBPs. An odor/pheromone is introduced, which competes with the 1-NPN occupation of the ligand binding slot of the OBP, causing a decrease in signal.

OBPs are robust enough to withstand wide pH ranges and temperatures (even 80–100 °C)^14^ without denaturing and losing their binding properties. In addition, OBPs are considerably smaller than olfactory receptors (ORs) because ORs are large integral membrane proteins. OBPs don’t need to be membrane-bound to assume their functional form. This makes OBPs ideal candidates for binding protein-derived probes mounted on biosensors, although the use of OBPs in such sensors has only been attempted in select studies^15^. Although there have been numerous OBP functional studies within the last two decades, no comprehensive database exists for OBP-VOC interactions. iOBPdb provides a novel platform for broadly studying odorant binding proteins and volatile organic compounds interactions. The iOBPdb:

- Provides for the first time a comprehensive dataset of compiled OBP-VOC binding interactions in the form of a consolidated and validated database.
- Provides a platform to compare multiple OBPs from different insect species, allowing robust comparative analysis.

## Methods

### Data gathering

OBP binding data was obtained through an extensive survey of the literature from the last 15 years (see the complete literature sources for the compiled data in the data reporting section). There have been 216 functional studies of 382 unique OBPs from 91 insect species. These functional studies surveyed over 600 potential binding molecules encoded by a CAS (Chemical Abstracts Service) number identifier. Structural information for these molecules was obtained by retrieving the respective molecular formula and SMILES (simplified molecular-input line-entry system) string associated with the CAS identifier through PubChem’s programmatic API, PUG REST^16^. Functional groups on these molecules were identified by analyzing the underlying SMILES text strings. Additional information was obtained from existing databases and literature, making it easier to identify potentially helpful OBPs or VOCs that may be pertinent to human health and biotechnological applications. This includes VOC profiles in healthy humans^17^ and VOC profiles of various human respiratory diseases^18–28^.

**Fig. 3:**
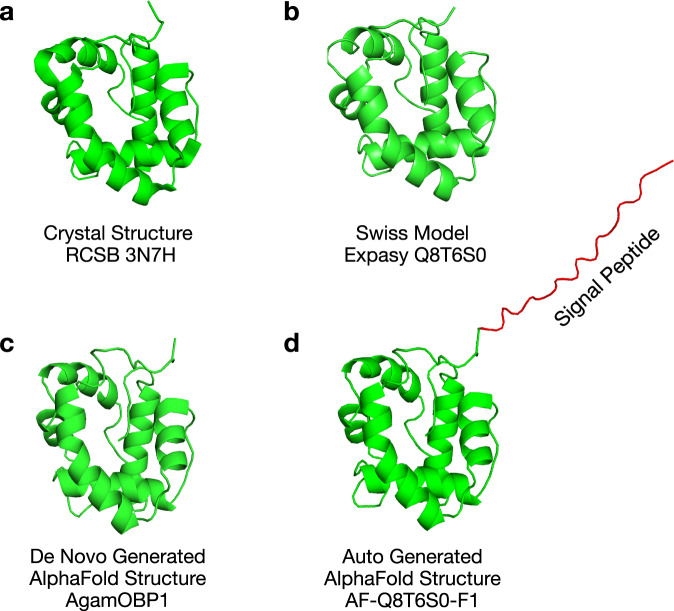
Various protein structures for AgamOBP1. (**a**) A fully resolved crystal structure was generated experimentally using X-ray diffraction and deposited in RCSB. (**b**) Predicted structure using homology matching via the Expasy Swiss Model server. Swiss Model ID is the same as the UniProt ID. (**c**) De novo generated structure using AlphaFold V2.0 using the cleaved OBP sequence. (**d**) Every OBP with an associated UniProt ID also has an accompanying auto generated AlphaFold V2.0 structure, which takes the whole protein with the signal peptide as an input. These structures are not in the dataset but can be found online using the UniProt ID of the OBP.

OBP structural data was also retrieved or generated for every OBP in the dataset. PDB (Protein Data Bank) entries that contained the structural information of specific OBPs were retrieved if fully resolved OBPs exist in RCSB (https://www.rcsb.org) or homology-matched OBPs exist on the Expasy Swiss-Model database (https://swissmodel.expasy.org). However, pre-existing structures only exist for some OBPs. To compensate for this, structures are de novo generated using AlphaFold V2.0 for all OBPs^29^. While there are auto-generated AlphaFold V2.0 structures available for some OBPs in the AlphaFold database (https://alphafold.ebi.ac.uk) and UniProt (https://www.uniprot.org/), we chose not to include them as they were generated using the entire protein sequence with the signal peptide still attached as opposed to cleaved protein sequence. The signal peptide does not confer any functional property associated with VOC binding. To obtain AlphaFold V2.0 structures for OBPs without the signal peptide required de novo generation, specifically using the ColabFold implementation provided by Mirdita *et al*.^30^. The differences between these structures are shown more clearly in Fig. 3. Should users still desire PDBs for the OBP structures with the signal sequences attached, they can search for them online using the UniProt accessions in the dataset for their OBP of interest.

## Data Records

### Database architecture and access

**Fig. 4:**
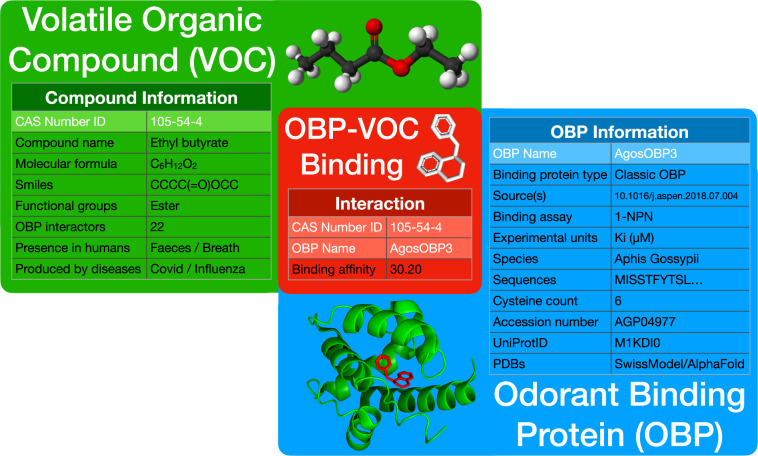
Organization of the iOBPdb dataset used to construct the many-to-many relational database. The full dataset consists of 3 separate CSV files. Compound_info.csv is a table containing information about all the volatile organic compounds in the database and is referenced using the key identifier CAS Number ID (shown in green). OBP_info.csv is a table containing information about all the odorant binding proteins in the database and is referenced using the key identifier OBP Name (shown in blue). Compound_OBP_binding.csv is a junction table containing the binding affinities of all recorded OBP-VOC interactions (shown in red).

A many-to-many relational database was modeled and designed to organize information collected from the literature search, which we have termed iOBPdb. A static archival version is stored on Zenodo, a public digital repository for depositing scientific data (10.5281/zenodo.7860280)^31^. A static version is also available on ResearchWorks, the University of Washington’s digital repository for scholarly works (http://hdl.handle.net/1773/49964)^32^. Figure 4 shows a schema for the full iOBPdb dataset, which consists of three separate CSV tables: Compound_info.csv, OBP_info.csv, and Compound_OBP_binding.csv.

### VOC information

The specific attributes of every volatile organic compound (VOC) are found in a data table stored on Compound_info.csv. The key identifier for each VOC is its CAS Number ID stored in the table’s first column. A CAS registry number is a unique identifier of three serial numbers separated by two hyphens, such as 105-54-4 in the case of ethyl butyrate, assigned for every molecule identified in the CAS chemical registry. Over 200 million unique organic and inorganic compounds are designated with a unique CAS registry number (https://www.cas.org/cas-data/cas-registry).

Compound name(s) are stored in Column 2, delimited with a slash “/”. These could be the compound’s IUPAC name, common names, or other names synonymous with the compound. Column 3 contains a precomputed number of OBPs that interact with the compound. Column 4 contains the molecular formula for the compound from which its molecular weight can be computed. Column 5 includes the compound’s SMILES, representing the compound’s atomic construction via ASCII string notation. The following 57 columns, columns 6–62, indicate if a functional group is present in the compounds with a “yes” and were determined by looking for functional group motifs in the underlying SMILES string via python’s RDKit package for cheminformatics. The following 7 columns, columns 63–69, describe if the compound is present in certain bodily fluids of healthy humans, with a “yes”, and were determined through literature search^17^. The final 17 columns, columns 70–86, describe if the compound is detectable and elevated in humans infected with respiratory diseases, with a “yes”, and was also determined through literature search^18–28^. If column information is unavailable for a particular row-wise compound, it is represented as “-” instead of an empty cell. Figure 4 summarizes this data table inside the green box showing the corresponding information of the VOC ethyl butyrate.

### OBP information

The characteristics and information pertinent to every odorant binding protein (OBP) are stored in a data table on OBP_info.csv. The key identifier for each OBP is its name stored in the table’s first column. The OBP name consists of three parts, the concatenated binomial name with the first capital letter denoting the genus and three subsequent lowercase letters indicating the species, the specific type of protein in all caps, and the number which indicates the order of discovery of that particular gene during genomic analysis. For example, the OBP name AgosOBP3 should be read as a combination of Agos, which comes from the species name Aphis gossypii, OBP, which specifies the binding protein type, and 3, which is the order that particular OBP gene was discovered in Aphis gossypii.

The binding protein type is specified in column 2, which classifies OBPs by specific properties such as sequence relatedness, cysteine count, etc. (OBP classification is further explained in the introduction). Columns 3–5 are records of the functional studies in which the specific OBP was characterized and include the study’s DOI source, publication date, and accompanying formal citation. Multiple sources are delimited with a “|”. Columns 6–7 contain the experimental binding assay used, and corresponding units used to measure the OBP’s binding affinity. Since this dataset is only focused on insect OBPs, the column for binding assay only contains 1-NPN, and the unit column only contains Ki in µM. The experimental standard for measuring insect OBP binding affinity is to employ a competitive binding assay, which uses 1-NPN as the fluorescent signaling molecule. Column 8 gives the species name that OBP was found in. Column 9 shows the complete amino acid sequence of the protein, and column 10 shows the cleaved sequence of the protein without its signaling peptide. The cleaved sequence describes the fully functioning protein. Column 11 contains the precomputed number of cysteines in the cleaved amino acid sequence. Column 12 provides the accession number associated with the protein’s amino acid sequence stored on GenBank, NIH’s repository for genetic and protein sequences (https://www.ncbi.nlm.nih.gov/genbank/). Column 13 provides the UniProt ID associated with the protein sequence if available on UniProt, a public database of protein sequences (https://www.uniprot.org/). Columns 14–16 indicate if PDB files describing the protein structure of the OBP are available for the specific protein. PDBs are obtained either from RCSB, a repository of curated and experimentally determined PDBs, or are generated through prediction using the Expasy Swiss-Model homology modeling server or AlphaFold’s AI system for protein structure prediction. If column information is unavailable for a particular row-wise OBP, it is represented as “-” instead of an empty cell. Figure 4 summarizes this data table inside the blue box showing the corresponding information of the OBP AgosOBP3.

### OBP-VOC binding information

The binding affinity of every recorded OBP-VOC interaction is found in a data table stored on Compound_OBP_binding.csv. Unlike the previously described data tables, which are simple column tables, the OBP-VOC binding data table is a junction table. The junction table is stored as a matrix of OBP-VOC interactions, with the matrix columns containing the key IDs of the OBPs and the matrix rows containing the key IDs for the VOCs. The units will always be Ki in µM, as discussed previously in the OBP information section. If binding data information is unavailable for a particular OBP-VOC pair, it is represented as “-” instead of an empty cell. Figure 4 summarizes this data table inside the red box showing the binding affinity for the specific interaction between the VOC ethyl butyrate and the OBP AgosOBP3.

### De Novo generated PDB structures of OBPs

Additionally, for each OBP in the dataset, an accompanying predicted AlphaFold protein structure is stored as a generated PDB file. The collection of all de novo generated PDBs is stored as a separate zip file titled: AlphaFold_Denovo_PDBs.zip. Each PDB structure was produced using the cleaved OBP amino acid sequence, feeding them as queries into ColabFold using all default settings for a typical monomer protein^30^. The output PDB with the best average pLDDT score was saved and titled simply as their OBP name, followed by “.pdb”. For example, the de novo generated AlphaFold PDB file for AgosOBP3 would be titled AgosOBP3.pdb in the zip file folder.

## Technical Validation

### Phylogenetic validation of database OBPs

**Fig. 5:**
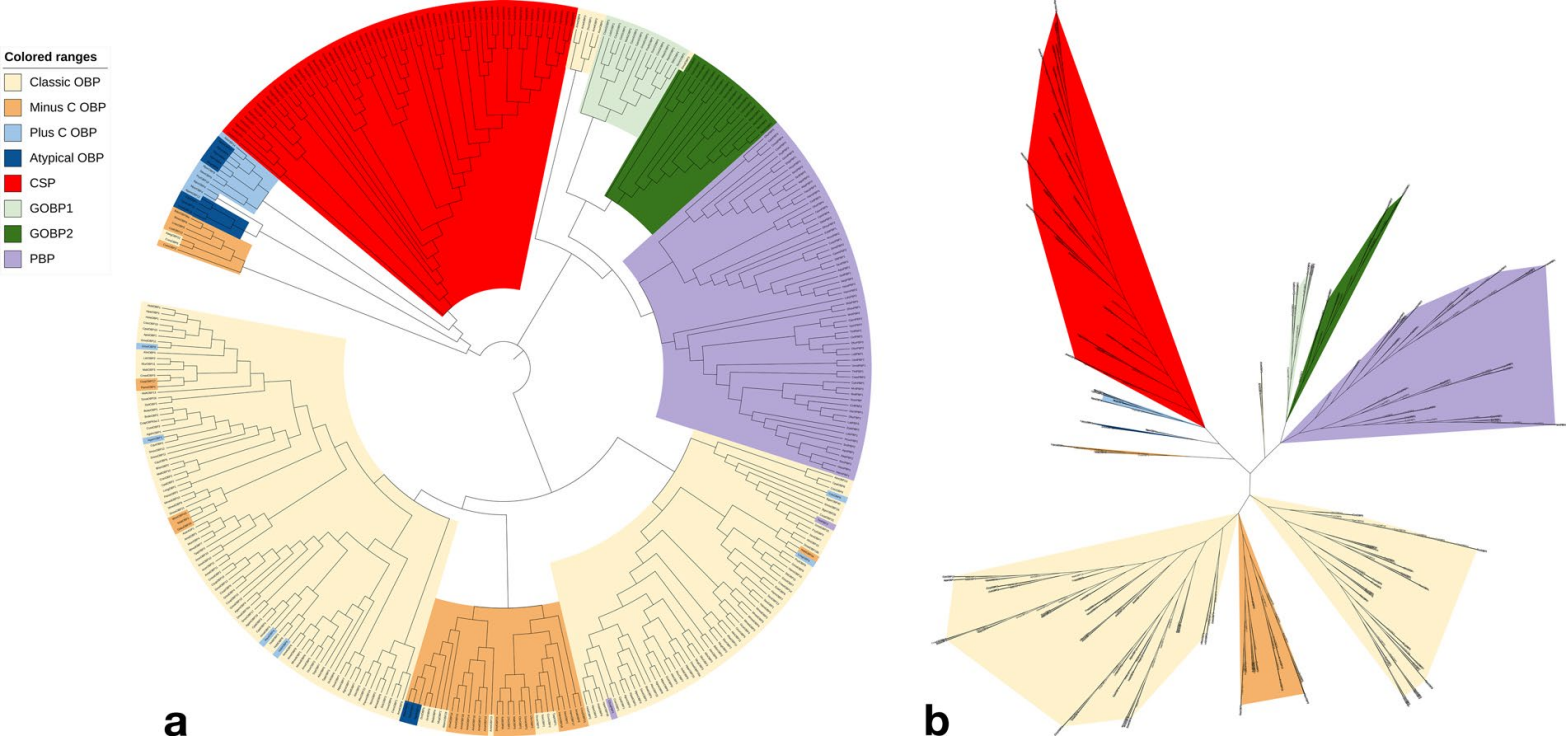
Phylogenetic representation of the insects OBPs: (**a**) Circular phylogenetic map, and (**b**) Unrooted phylogenetic map using 382 unique OBP sequences.

OBP amino acid sequences were typically directly provided in the texts of the functional studies or indirectly through their associated NCBI GenBank accession number. It is challenging to tell superficially whether the provided sequence belongs to their professed OBP type/subfamily. To validate whether the collected OBP sequences cluster according to their familial similarity, we constructed a phylogenetic map for all OBPs in the database in various schemes to validate the relative similarities of the structure-function relationships among the OBPs from insects. The Newick tree was generated using Clustal Omega multiple sequence alignments (http://www.ebi.ac.uk/Tools/msa/clustalo/)^33^. The resulting phylogenetic map was constructed and displayed using the iTOL web server^34^. The results of this phylogenetic analysis can be seen in its circular form in Fig. 5a and unrooted form in Fig. 5b.

Despite sharing similar cysteine counts, CSPs form a unique clade separate from the minus-C OBPs. Additionally, one can observe two distinct subfamilies of minus-C OBPs, one more closely related to CSPs and the other more closely related to classical OBPs. A distinct sub-family of atypical OBPs is adjacent to the small sub-family of plus-C OBPs. However, we generally observe less sequence similarity amongst plus-C and atypical OBPs as they are more generally scattered across the phylogenetic tree. Perhaps this is due to the dataset’s low coverage of sequences associated with plus-C and atypical OBPs.

On the other hand, the GOBPs and PBPs form a very distinct monophyletic clade separate from CSPs and OBPs. GOBPs also clearly subdivide into two families termed groups 1 and 2. The classic OBPs also generally cluster with other classic OBPs. From this, we can conclude that the vast majority of the given sequences for OBPs in the dataset cluster in accordance with their associated type/subfamily, i.e., CSPs, Minus-C, Plus-C, atypical, GOBPs, PBBs, and classic OBPs. There are some examples of sequences that did not cluster with their associated sub-family as would be expected. TintPBP3 (GenBank accession ID: AXF80671) and CbuqPBP1 (GenBank accession ID: AOF39987) did not cluster with the other PBP sequences in the dataset. We checked the GenBank sequences with our dataset sequences to ensure correspondence, which there was. These sequences may be initially interpreted as PBPs for other reasons, such as their binding profiles, even though they share minimal sequence similarity with the other dataset PBP sequences.

### Corroborated OBP molecular weights by subfamily

**Fig. 6:**
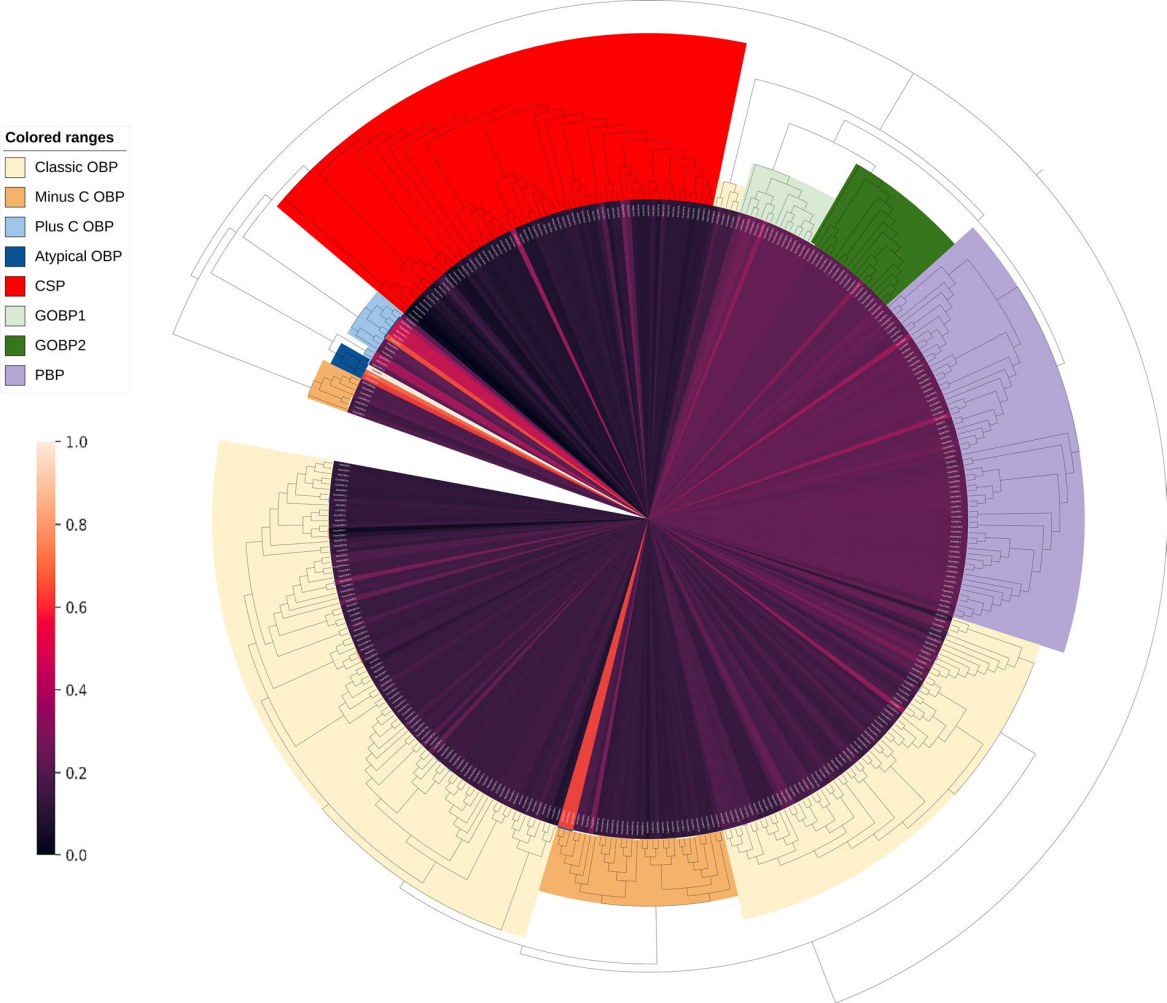
Distribution of normalized OBP molecular weights overlayed with the phylogenetic tree of OBPs. The molecular weights for each OBP were determined using a cleaved OBP sequence without the signal peptide.

The heatmap in Fig. 6 that overlays the phylogenetic map showcases the distribution of molecular weights of all OBPs. The molecular weight of the OBPs is directly related to the length of their underlying amino acid sequence. This can be more clearly seen in Table 1.

**Table 1:** Mean Sequence Length and Molecular Weights of OBPs by Subfamily.

OBP Subfamily	Amino Acid Sequence Length (with signal peptide)		Amino Acid Sequence Length (without signal peptide)		Molecular Weight of Cleaved Protein (kDa)	
	mean	standard deviation	mean	standard deviation	mean	standard deviation
Classic OBP	144.09	9.64	124.75	8.87	14.09	1.05
Minus C OBP	136.83	9.91	119.27	8.64	13.58	1.14
Plus C OBP	174.56	38.24	156.31	37.44	17.44	3.87
Atypical OBP	230.33	40.98	214.89	43.87	24.68	5.17
CSP	127.99	12.56	109.37	12.29	12.70	1.41
GOBP1	167.20	4.33	146.53	5.97	17.15	0.65
GOBP2	160.89	2.97	142.67	5.11	16.31	0.57
PBP	163.92	7.48	143.47	5.75	16.24	0.70

Figure 6 and Table 1 show that CSPs are the smallest subfamily of proteins, whereas the plus-C and atypical proteins are the largest. GOBPs and PBPs are similar in size and consistently larger than the classical OBPs. There is also no discernable difference in size between minus-C OBPs and classic OBPs. However, minus-C OBPs are considerably larger than CSPs despite both sharing a similar number of cysteines.

These results are in line with the literature and expectations. For comparison, an OBP review paper by Fan *et al*. gives the following expected molecular weights: Classic OBPs ~ 14 kDa, Plus-C OBPs ~17–25 kDa, and atypical OBPs up to 38 kDa^35^. The mean molecular weights in the dataset, as shown in Table 1, are 14.09 kDa for Classic OBPs, 17.44 kDa for Plus-C OBPs, and 24.68 kDa for atypical OBPs (the largest atypical OBP in the dataset being over 33 kDa). In a review of moth PBPs and GOBPs by Guo *et al*., they state that PBPs and GOBPs are typically around “15.89–17.17 kDa (without the signal peptide),” which also corresponds with the molecular weight values reported in Table 1 for PBPs and GOBPs^36^. In a review paper on insect OBPs and CSPs by Xu *et al*., they state, “Insect CSPs are smaller than OBPs with about 100–120 amino acid,” which aligns with the mean of 109.37 amino acids for CSP sequences in the dataset^37^.

It should be noted that while there is general agreement between the dataset values and literature values for OBP size, there are examples of extreme outliers that do not fit neatly with their associated OBP subfamily. This is reflected in the significant standard deviation of some OBP subfamilies. These are still valid sequences even if they break the conventional paradigm, as OBPs naturally are a diverse and divergent family of proteins. Their underlying sequence similarity can vary significantly amongst different species, even within the same species, and can be as low as 20%^38^. This is an expected property of OBP sequences.

## Usage Notes

### iOBPdb web portal

The iOBPdb database is also accessible through a web portal (www.iobpdb.com). Information pertinent to OBP-VOC binding interactions is accessible through searching key terms or browsing a directory of Compounds or OBPs. Selecting a Compound ID or OBP name will direct you to a corresponding information page containing the pertinent data. Database information is downloadable most easily from the Download data files page. The Download data files page contains a CSV of all VOC information (Compound_info.csv), a CSV of all available OBP information (OBP_info.csv), a CSV of all OBP-VOC binding information (Compound_OBP_binding.csv.), as well as a zip file containing all de novo generated PDB structures available on the website (AlphaFold_Denovo_PDBs.zip.). A complete site rip is also available as a zip file containing all the above information (iOBPdb_full_site.zip).

## Data Availability

iOBPdb GitHub source code can be accessed online here: https://github.com/sshuklz/iobpdb_app.
